# THE ASSOCIATION BETWEEN DIGITAL TECHNOLOGY ACTIVITIES AND COGNITIVE DOMAINS OF COMMUNITY DWELLING OLDER ADULTS

**DOI:** 10.1093/geroni/igae098.3898

**Published:** 2024-12-31

**Authors:** Erh-Chi Hsu, Eric Jutkowitz, Kali Thomas

**Affiliations:** Johns Hopkins University, Baltimore, Maryland, United States; Brown University, Brown University, Rhode Island, United States; Johns Hopkins University, Baltimore, Maryland, United States

## Abstract

While prior research has established a relationship between digital technology use and improved cognitive function among older adults, the impacts of different technology activities on specific cognitive domains remain underexplored. This study examines associations between various technology activities and cognitive trajectories in community-dwelling older adults without dementia. We utilized repeated cross-sectional data from 5,596 participants in the National Health and Aging Trends Study (2015-2022). Technology activities included online shopping, banking, medication refills, social network site visits, and checking health conditions online. Cognitive domains assessed were episodic memory, executive function, and orientation. Covariates included sex, age, race, education, living arrangement, income, daily and instrumental activity difficulties, self-rated health, rurality, and device ownership. Survey-weighted mixed-effect models with random intercepts evaluated the associations between technology activities and cognitive trajectories, with descriptive stratification by sex. In 2015, online banking (28%) and visiting social network sites (25%) were the most popular activities, with females generally showing lower usage except for social network visits (F: 27%; M: 23%). Social engagement online had the strongest positive association with episodic memory (β=0.50, 95% CI: 0.33-0.67, p< 0.001) and orientation (β=0.15, 95% CI: 0.10-0.21, p< 0.001) independent of time. Online banking showed the strongest positive association with executive function (β=0.12, 95% CI: 0.06-0.18, p< 0.001) independent of time. Notably, all five activities were more beneficial for females regarding episodic memory (p< 0.001). These findings suggest that interventions promoting online instrumental activities (shopping, banking, medication refills) and social engagement online could enhance cognitive function in older adults, particularly females.

